# Factors associated with intention to exclusive breastfeed in central women’s hospital, Yangon, Myanmar

**DOI:** 10.1186/s13006-017-0120-2

**Published:** 2017-07-06

**Authors:** Myat Pan Hmone, Mu Li, Kingsley Agho, Ashraful Alam, Michael J. Dibley

**Affiliations:** 1 Sydney School of Public Health, The University of Sydney, Sydney, NSW 2006 Australia; 2 School of Science and Health, Western Sydney University, Penrith, NSW 2571 Australia

**Keywords:** Intention to breastfeed, Exclusive breastfeeding, Associated factors, Socio-economic factors, Breastfeeding knowledge, Sources of breastfeeding, Pregnant women, Myanmar

## Abstract

**Background:**

Under-nutrition is a public health problem in Myanmar. Despite current efforts, the exclusive breastfeeding rate (EBF) for children under six months is only 24%. Intention to breastfeed is a strong predictor for long-term breastfeeding, however, little is known about pregnant women’s breastfeeding intentions in Myanmar. We, therefore, aimed to identify the factors associated with women’s intention to EBF.

**Method:**

Data in this article was collected in a baseline survey for a randomized controlled trial, which aimed to assess the impact of mobile text messages on the breastfeeding practices of women in Yangon, Myanmar. A total of 353 pregnant women at 28–34 weeks of gestation, recruited into the trial from the antenatal clinics of the Central Women’s Hospital, Yangon, Myanmar, responded to the baseline survey questions, which included background information and breastfeeding related characteristics. To determine factors associated with women’s intention to EBF logistic regression was used to analyse individual demographic, household economic and breastfeeding characteristics. In-depth interviews were performed with a sub-sample of 24 women who participated in the survey, to gain a further understanding of these associated factors.

**Results:**

After adjusting for potential confounders, working women were less likely to intend to EBF (adjusted odds ratio (AOR) = 0.30, CI 0.17–0.53). Women from rich households (AOR = 2.43, CI 1.08–5.47) and middle income households (AOR = 1.79, CI 1.01–3.16); those who had high (AOR = 10.19, CI 3.43–30.23) and medium (AOR = 5.46, CI 1.79–16.72) breastfeeding knowledge levels, and received information from health professionals (AOR = 2.29, CI 1.29–4.03) and mobile internet (AOR 3.62, CI 2.04–6.41) had a higher intention to EBF. These findings were supported by qualitative analysis, which revealed that returning to work was the main barrier; health staff and printed media are reliable sources and; women with higher knowledge had high intentions to EBF.

**Conclusions:**

EBF intention was influenced by many factors. Breastfeeding promotion programs should target the poor, working women and women with lower breastfeeding knowledge. Breastfeeding education via health staff and the Internet, breastfeeding facilities at the work place and longer maternity leave in the private sector should all be encouraged.

**Trial registration:**

Australian New Zealand Clinical Trials Registry ACTRN12615000063516.

## Background

World Health Organization (WHO) recommends that all infants should be exclusively breastfed (EBF) in the first six months of life [1]. Several studies acknowledge that breastfeeding is an ideal food for healthy growth and development of infants, serves as a key protective factor against common childhood infectious diseases, and has short and long term benefits for children and mothers [2–5]. Decisions and breastfeeding practices are influenced by a wide range of individual, cultural and socioeconomic factors [6–10]. Studies show that awareness, previous experiences, perceived barriers and self-efficacy for breastfeeding all influence women’s actual breastfeeding practices [9, 11, 12].

In Myanmar, breastfeeding is practiced universally and although 90% of mothers were aware of breastmilk benefits and 76% fed breastmilk to their newborn within one hour after birth, only 24% of babies under six months of age were EBF [13]. Myanmar has the second lowest EBF rate in South-East Asian countries, behind Thailand [14, 15]. Undernutrition is the underlying cause of estimated 45% of deaths among under-five children in middle and low income countries including Myanmar [16]. Government reported that 23% of under-five children in Myanmar were underweight and 35% were stunted [13], which exceeded the global average of 25% [17]. The high stunting rate in Myanmar might contribute to the high infant and under-five child mortality rates in Myanmar (41 and 52 deaths per 1000 live births) [15]. Though the government has made several efforts to improve infant feeding practices in Myanmar, it is a challenge to find reliable data to inform the health program design.

Intention to breastfeed is an important predictor of actual breastfeeding practices, and if a woman intends to breastfeed before delivery, she will have a higher chance of exclusively breastfeeding and maintaining the practice even if challenged. A very strong desire to breastfeed was found to have a positive association with breastfeeding at 6 months in a study by Forster et al. [18]. This finding was supported by a review by Meedya et al. which found that woman’s breastfeeding intention, her breastfeeding self-efficacy and her social support were positively associated with breastfeeding duration [19]. Likewise, studies conducted in Australia [18], United Kingdom [20] and USA [21] have shown that pregnant women’s pre-birth breastfeeding intention was a good predicator of the duration of their breastfeeding.

However, no similar studies have been conducted in Myanmar to explore pregnant mothers’ intention to EBF and most available studies were conducted with postpartum women. The literature related to breastfeeding, which was most commonly found, was about the early initiation of breastfeeding, and knowledge, attitudes and awareness about breastfeeding, with few studies on intentions for breastfeeding. A more detailed understanding of the factors associated with pregnant women’s intention to EBF is needed to assist policy makers develop effective interventions and to improve the rates of EBF in Myanmar. This paper aims to identify the factors associated with women’s intention to EBF in Myanmar and qualitative study aims to give a deeper understanding of the associated factors.

## Methods

### Study design

The data for this study was collected in a baseline survey for a randomized controlled trial [22], which aimed to assess the impact of mobile text messages on the breastfeeding practices of women enrolled through the antenatal clinic, Central Women’s Hospital, Yangon, Myanmar. This study combines quantitative cross sectional survey data and qualitative in-depth interviews. A total of 353 pregnant women at 28–34 weeks of gestation, who were recruited into the trial, responded to the baseline survey questions over 6 weeks from January to February 2015. The survey collected data about factors potentially associated with the pregnant women’s intention to EBF during study period. Qualitative study was also performed with a sub-sample of surveyed women to gain a deeper understanding of the associated factors after the survey data collection.

The Central Women’s Hospital, Yangon was purposively selected because it is the largest tertiary public hospital in Myanmar providing free quality women’s health services, and was an ideal setting where we could recruit women from various walks of life. It is accredited as a baby friendly hospital and women received a brief breastfeeding education during visits. This study was a part of a two arm randomized controlled trial, which aimed to investigate the effectiveness of mobile text messages on the breastfeeding practices of women from intervention and control groups [22–24].

### Study variables

**Fig. 1:**
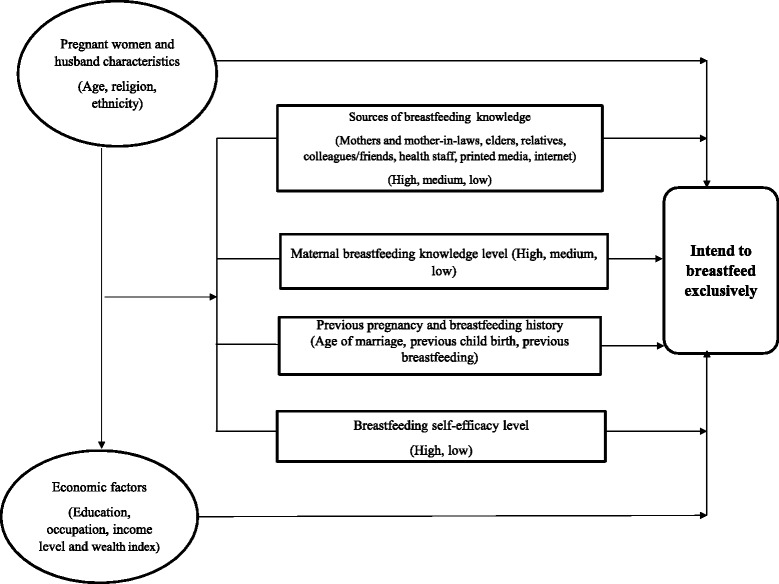
Conceptual framework for factors associating women’s intention to exclusive breastfeeding; The selection of potential determinants of intention to EBF was guided by the conceptual framework. The independent variables were ‘the pregnant woman and her husband’s characteristics (age, religion, ethnicity)’; ‘economic factors (education, occupation, income level and wealth index)’; and ‘sources of breastfeeding knowledge’, ‘maternal breastfeeding knowledge level’, ‘previous pregnancy and breastfeeding history’, ‘breastfeeding self-efficacy level’. Dependent variable was ‘women’s intention to exclusive breastfeeding’

The dependent variable was the women’s intention to EBF and we use WHO definition of EBF [25]. The selection of potential determinants of intention to EBF was guided by the conceptual framework described in Fig. 1. The independent variables were the pregnant woman and her husband’s characteristics (age, religion and ethnicity); economic factors (education, occupation, income level and wealth index); and breastfeeding related characteristics (previous experiences, knowledge and sources, and self-efficacy). We used a 33-item breastfeeding self-efficacy scale with a five level Likert scale [11, 26]. Household wealth index and breastfeeding knowledge levels were grouped into low/medium/high categories. As recommended by the World Bank Poverty Network [27], the wealth index was constructed using principal component analysis to determine

the weights of the index based on information collected about several household characteristics and household assets. The household received 0 or 1 score depending on the possession of household assets, such as electricity, radio, television. The total scores were grouped into poor (bottom 40%), middle (next 40%) and rich (the top 20%). There were 9 breastfeeding knowledge questions and knowledge on breastmilk benefits, colostrum and recommended practice were assessed. Women received 0 for the wrong response and 9 for all correct answers. Breastfeeding self-efficacy scales were ranged from 33 to 165, with higher scores indicating higher levels of breastfeeding self-efficacy. Women who scored 0 to 82 positioned as low level and 83 to 165 as high level.

### Sample size

The sample size for this study was determined by the requirements of the randomized controlled [22] for which it was the baseline survey. Sample size for the trial was calculated with the assumptions of 80% power, 5% two-sided alpha and 13% expected loss at follow-up. The EBF rate of Myanmar children at six months of age was 15% [4] and we hypothesized that EBF in the intervention group will be increased two-fold. The estimated sample size was 312 (156 per group) and the final sample size requirement was 353 participants to allow for 13% loss at follow-up. We estimate that for the present study this sample size would provide 80% power to detect a 30% relative difference (p1 0.3525, p2 0.5000) in a risk factor between women who intended to exclusively breast feed versus women who did not. Therefore, sample size for the submitted study was 353 with power = 0.80. STATA version 13.0 (Stata Corporation, USA) was used for sample size estimation and data analysis.

### Quantitative part of the study

#### Participants

We sampled all eligible pregnant women visiting the antenatal clinics of the Central Women’s Hospital, Yangon during the study period. Inclusion criteria were pregnant women who were 28 to 34 weeks of gestation, had an uncomplicated and singleton pregnancy by ultrasound, had no illness that would contradict breastfeeding, were able to read and write Myanmar language, had access to a mobile phone and lived in an area with mobile network coverage. Exclusion criteria were having pregnancy complications, a multiple pregnancy and known medical condition(s) including mental illness that might hinder breastfeeding. We excluded women who declined to participate in the study. A total of 353 women were recruited for the study.

#### Data collection

The antenatal care clinic operates Monday to Friday and from 8 am to 12 pm. During data collection period, participant eligibility was assessed via hospital attendance registry (used by hospital staff) and antenatal care records (kept by the participant) in which information such as age, weeks of gestation, pregnancy assessment and medical history were recorded. Researchers identified potential participants with the help of hospital nurses, approached potential eligible women at antenatal clinic waiting area, explained the study nature and confirmed eligibility. If a woman was agreed to participate, a written informed consent was obtained and survey data was collected with a tablet using a Dimagi CommCare application [28]. We could recruit average 10–15 women per day as most women visited hospital after 34 weeks of gestation and some declined to participate.

The survey questionnaires had 7 sections, including individual socio-economic status; previous breastfeeding history; breastfeeding knowledge and sources; perceived breastfeeding self-efficacy, intended breastfeeding patterns and reasons given. In developing the survey questionnaires, we adapted questions from a number of sources [9, 13, 29, 30]. Intention to breastfeed was measured by asking the woman ‘Do you plan to breastfeed your child when you deliver?’, and a subsequent question, ‘Do you plan to exclusively breastfeed your child?’ while explaining the meaning of EBF. If a respondent answered ‘Yes’ a further question was asked about the age to which she planned to exclusively breastfeed her child.

#### Data analysis

STATA version 13.0 (Stata Corporation, USA) [31] was used for data analysis. Independent variable was examined against a set of independent variables to determine the prevalence and factors associated with intention to EBF. Preliminary analyses involved frequency tabulations of all selected characteristics. The associations were examined using 95% confidence intervals around the prevalence estimates and chi square tests.

Univariate and multivariate logistic regression were conducted to determine the unadjusted and adjusted odds ratios of intention to EBF.A six-stage model guided by the conceptual framework was constructed in the multivariate regression analysis. In the first model, the individual factors were entered into the baseline multivariable model and a manual stepwise backwards elimination process was conducted and only variables significantly associated with intention to EBF at a 0.05 significance level were retained in the model (model 1). Second, household economic factors were entered into model 1, and factors with *p*-values <0.05 were retained (model 2) after a backwards elimination process. Similar procedures were performed for subsequent models (significant factors from sources of knowledge were retained into model 3 and significant factors from knowledge levels were retained into model4) and finally previous pregnancy and breastfeeding history factors and breastfeeding self-efficacy levels were entered into the fifth and sixth stages, respectively. The odds ratios (OR) and their 95% confidence interval were used to measure the level of association of the factors with intention to EBF.

### Qualitative part of the study

#### Participants

We used purposive sampling, and selected a sub-sample of 24 women who participated in the baseline survey. Women were eligible to participate if they agreed to spend one hour after the survey and we recruited maximum 2 women per day. We pretested the interview guidelines with three pregnant women prior to data collection and in-depth interviews were conducted using these guidelines.

#### Data collection

After taking consents for interviews and audio-recordings, in-depth interviews were conducted immediately after the survey at a room situated at the hospital clinic. We explored topics such as perceptions and awareness of the benefits of breastmilk and recommended breastfeeding practice, sources of information and feedback on health staff, self-efficacy to breastfeed and perceived barriers to practice EBF for 6 months.

#### Data analysis

Thematic analysis was used [32]. All recorded interviews were transcribed verbatim in Burmese, saved in a word document and checked for accuracy. MPH translated a sample of the transcripts into English, MPH and AA read and discussed two transcripts and prepared a draft code list. MPH then added thematic codes emerged from a thorough reading of the transcripts and the final code list was discussed among the authors to increase inter-coder reliability.

## Results

The study used a combination of quantitative survey data collection and qualitative in-depth interviews.

### Quantitative study findings

**Fig. 2:**
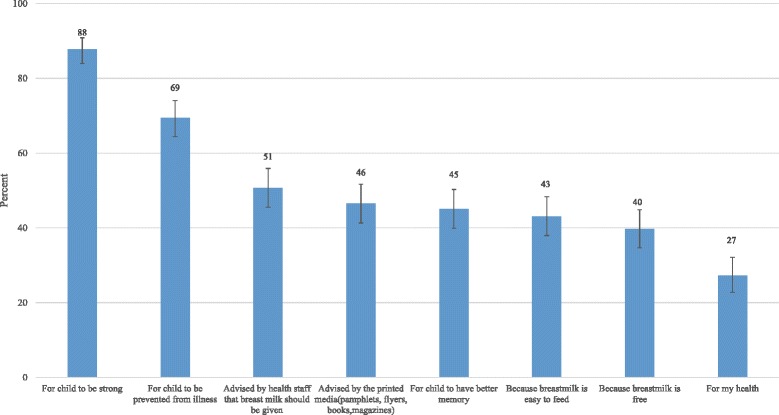
Mother’s reasons for exclusive breastfeeding intention; reasons given by mothers for their intention to exclusive breastfeeding were assessed by a multiple response question and presented in ‘x’ axis. Reasons given were ‘for child to be strong’, ‘for child to be prevented from illness’, ‘for child to have better memory’, ‘be advised by health staff that breastmilk should be given’, ‘be advised by printed media’, ‘because breastmilk is easy to feed’, ‘because breastmilk is free’ and ‘for my health’. Y axis shows ‘percentage’

Figure 2 describes reasons for women’s intention to EBF. Reasons given by the respondents were ‘for child to be strong’, ‘for child to be prevented from illness’, ‘for child to have better memory’, ‘be advised by health staff that breastmilk should be given’, ‘be advised by printed media’, ‘because breastmilk is easy to feed’, ‘because breastmilk is free and good for my health’. The most commonly given reasons were ‘for child to be strong (88%)’, ‘for child to be prevented from illness (69%)’ and ‘be advised by health staff that breastmilk should be given’ (51%)’. Table 1 shows that if a woman had high socio-economic status, had high breastfeeding knowledge or self-efficacy scale, or either her husband or herself was Bamar, she had high intention to EBF. Women who received knowledge from either health staff, printed media or internet had a significantly higher chance to practice intention to EBF than those who did not.

**Table 1:** Influence of individual, household and other breastfeeding related characteristics on women’s intention to breastfeed exclusively

Intention to breastfeed exclusively				
Variable	%	Number^a^	95% CI	*P*
Pregnant women’s characteristics				
Age (years)				
15–24	65.3	62	[55.14,74.17]	0.997
25–34	64.3	128	[57.39,70.70]	
35+	64.4	38	[51.44,75.56]	
Ethnicity				
Bamar	62.1	187	[56.49,67.46]	0.020*
Others	78.9	41	[65.63,87.92]	
Husbands’ characteristics				
Age (years)				
15–24	65.9	27	[50.20,78.67]	0.621
25–34	62.4	123	[55.44,68.95]	
35+	67.8	78	[58.73,75.75]	
Ethnicity				
Bamar	62.1	190	[56.50,67.38]	0.012*
Others	80.9	38	[67.05,89.76]	
Economic factors				
Women’s education				
Primary	66.7	10	[40.48,85.47]	0.518
Secondary	62.5	145	[56.06,68.52]	
University	68.9	73	[59.41,76.98]	
Women’s occupation				
Non-working	70.0	177	[64.00,75.32]	<0.001***
Working	51.0	51	[41.24,60.68]	
Husbands’ education				
Primary	57.5	23	[41.88,71.75]	0.317
Secondary	63.5	141	[56.95,69.61]	
University	70.3	64	[60.13,78.84]	
Husbands’ occupation				
Non-working	100.0	2		0.293
Working	64.3	226	[59.21,69.25]	
Household wealth index				
Poor	56.3	80	[48.04,64.29]	0.006**
Middle	66.4	99	[58.45,73.59]	
Rich	79.0	49	[67.09,87.45]	
Pregnancy and breastfeeding history				
Age of marriage				
15–24	65.1	134	[58.26,71.28]	0.800
25–34	63.2	86	[54.79,70.94]	
35+	72.7	8	[41.28,91.00]	
Previous birth history				
Yes	64.0	89	[55.69,71.60]	0.859
No	65.0	139	[58.29,71.08]	
Previous breastfeeding history				
Yes	63.9	85	[55.31,71.70]	0.89
No	66.7	4	[26.4,91.77]	
Breastfeeding knowledge level^b^				
Low	15.2	5	[6.42,31.72]	<0.001***
Medium	48.2	41	[37.80,58.83]	
High	77.5	182	[71.63,82.36]	
Breastfeeding knowledge sources				
Mothers and mother-in laws				
No	66.9	95	[58.72,74.17]	0.457
Yes	63.0	133	[56.29,69.31]	
Elders				
No	67.4	87	[58.86,74.99]	0.396
Yes	63.0	141	[56.40,69.05]	
Relatives				
No	68.1	192	[62.39,73.29]	0.006**
Yes	50.71	36	[39.19,62.14]	
Colleagues/friends				
No	67.0	132	[60.11,73.24]	0.287
Yes	61.5	96	[53.64,68.87]	
Health staff				
No	53.0	107	[46.04,59.79]	<0.001***
Printed media				
No	56.0	135	[49.66,62.19]	<0.001***
Yes	83.0	93	[74.88,88.93]	
Mobile internet				
No	45.3	77	[37.94,52.86]	<0.001***
Yes	82.5	151	[76.28,87.38]	
Breastfeeding self-efficacy scale^c^				
Low	47.8	22	[33.89,62.11]	0.011*
High	67.1	206	[61.62,72.15]	

^a^Number of pregnant women who had intention to exclusive breastfeeding (*n* = 222 out of 353 women)

^b^Breastfeeding knowledge, such as knowledge on breastmilk benefits, colostrum and recommended duration, was assessed by 9 questions and for each question, women received 0 for the wrong response and 1 for correct answer. Knowledge levels were categorized into Low (score 1–3), Medium (score 4–6) and High (score 7–9)

^c^Measuring breastfeeding self-efficacy with 33 items. Total scores range from 33 to 165, with higher scores indicating higher levels of breastfeeding self-efficacy. Scores levels were categorized into Low (score 1–82), and High (score 83–165)

CI, confidence interval

*p < 0.05; **p < 0.01; *** *p* < 0.001 by chi-squared test

In contrast, working mothers and women who received knowledge from relatives were less likely to have intention to EBF. The intended breastfeeding pattern did not differ significantly with the remaining characteristics such as age, religion, education, income, age of marriage, previous birth and breastfeeding histories and other sources of knowledge.

Table 2 presents the factors significantly associated with women’s intention to EBF with both unadjusted and adjusted odds ratios and confidence intervals. After adjusting for all other confounders, working mothers were less likely to intend to EBF compared with non-working mothers (adjusted OR (AOR) =0.30, 95% CI, 0.17, 0.53). Compared to poor households, the odds of the women’s intention to EBF was higher among rich households (AOR = 2.43, 95% CI 1.08–5.47) and medium (AOR = 1.79, 95% CI 1.01–3.16) level households. An increase in breastfeeding knowledge level was associated with intention to EBF with women with highest knowledge levels having odds of intent to EBF 10 times higher than women with lowest knowledge level (AOR = 10.19, 95% CI 3.43–30.23), and women with medium knowledge level had 5 times higher odds to have intention to EBF (AOR = 5.46, 95% CI 1.79–16.72) than those with low knowledge. Women who reported their sources of knowledge as health staff had twice the odds of intent EBF (AOR = 2.29, 95% CI 1.29–4.03) compared to women who did not. Users of mobile internet had odds approximately three times higher to intend to EBF (AOR 3.62, 95% CI 2.04–6.41) compared to women without mobile internet. Breastfeeding self -efficacy scale was not significantly associated with the women’s intention to EBF.

**Table 2:** Risk factors for pregnant women who intend to exclusive breastfeeding -unadjusted and adjusted Odds Ratio

Characteristics	Exclusive breastfeeding					
	Unadjusted			Adjusted		
Variable	OR^a^	95% CI	*P*	OR	95% CI	*P*
Pregnant women’s characteristics						
Age (years)						
15–24	1					
25–34	0.89	[0.50, 1.60]	0.697			
35+	0.72	[0.31,1.70]	0.456			
Ethnicity						
Bamar	1					
Others	1.76	[0.50,6.16]	0.380			
Husbands’ characteristics						
Age (years)						
15–24	1					
25–34	0.91	[0.44,1.87]	0.800			
35+	1.22	[0.56,2.64]	0.610			
Ethnicity						
Babmar	1					
Others	2.20	[0.63,7.63]	0.215			
Economic factors						
Women’s education						
Primary	1					
Secondary	0.63	[0.20,1.93]	0.416			
University	0.73	[0.22,2.45]	0.611			
Women’s occupation						
Non-working	1			1		
Working	0.42	[0.26,0.68]	<0.001***	0.30	[0.17,0.53]	<0.001***
Husband’s education						
Primary	1					
Secondary	1.56	[0.65,3.75]	0.324			
University	1.54	[0.57,4.12]	0.393			
Household wealth index						
Poor	1			1		
middle	1.66	[1.02,2.71]	0.041*	1.83	[1.01,3.16]	0.045*
Rich	3.17	[1.56,6.44]	0.001**	2.50	[1.08,5.47]	0.033*
Pregnancy and breastfeeding history						
Yes	1					
No	1.16	[0.20,6.56]	0.869			
Breastfeeding knowledge level^b^						
Low	1			1		
Medium	5.22	[1.84,14.80]	0.002**	5.46	[1.79,16.72]	0.003**
High	19.23	[7.08,52.25]	<0.001***	10.19	[3.43,30.23]	<0.001***
Breastfeeding knowledge sources						
Mothers and mother-in laws						
No	1					
Yes	0.98	[0.59,1.63]	0.944			
Elders						
No	1					
Yes	1.41	[0.82,2.41]	0.217			
Relatives						
No	1					
Yes	0.86	[0.46,1.60]	0.632			
Colleagues/friends						
No	1					
Yes	0.79	[0.47,1.32]	0.371			
Health staff						
No	1			1		
Yes	2.70	[1.61,4.54]	<0.001***	2.29	[1.29,4.03]	0.004**
Printed media						
No	1					
Yes	1.78	[0.94,3.38]	0.078			
Internet						
No	1			1		
Yes	4.79	[2.91,7.89]	<0.001***	3.62	[2.04,6.41]	<0.001***
Breastfeeding self-efficacy scale^c^						
Low	1					
High	2.23	[1.19,4.16]	0.012*			

^a^Odds Ratio (OR)

^b^Breastfeeding knowledge, such as knowledge on breastmilk benefits, colostrum and recommended duration, was assessed by 9 questions and for each question, women received 0 for the wrong response and 1 for correct answer. Knowledge levels were categorized into Low (score 1–3), Medium (score 4–6) and High (score 7–9)

^c^Measuring breastfeeding self-efficacy with 33 items. Total scores range from 33 to 165, with higher scores indicating higher levels of breastfeeding self-efficacy Scores levels were categorized into Low (score 1–82), and High (score 83–165)

CI, confidence interval

*p < 0.05; **p < 0.01; *** *p* < 0.001 by chi-squared test

### Qualitative study findings

The interviews generated 7 themes about pregnant women’s intention to EBF. Table 3 lists the themes and cites quotes for each theme. Women generally had high awareness of the benefits of breastmilk and mentioned benefits as ‘good for child’s physical health’ followed by ‘better memory’ while none of them mentioned breastmilk as ‘benefits to mothers’. Recommended duration of breastmilk was mentioned correctly as 6 months. Apparently, women with higher breastfeeding knowledge were more eager to practise EBF than women with low knowledge (Table 3, themes 1 and 2). All women planned to breastfeed and the majority intended to EBF for approximately 3 to 5 months. All women perceived work was the main obstacle to EBF due to not enough maternity leave especially in private sector and the lack of breastfeeding supporting environment (Table 3, theme 3). If a woman had worries about the quantity of breastmilk flow, she could not decide her intended feeding pattern during the interview. Some women told stories about ‘painful mastitis’ experienced from a previous pregnancy and were willing to give breastmilk only if they did not suffer pain (Table 3, theme 4).

**Table 3:** Themes and selected supporting quotes about factors associated with women’s intention to exclusive breastfeeding (extracted from in-depth interviews with pregnant women)

Themes	Selected quotes
1. Majority of the women were aware of the benefits of breastmilk and duration to practice EBF, and physical health was mentioned as a main benefit.	“I will give breastmilk for my child’s sake. I heard it prevents diarrhea and illness. Child could be strong, healthy and intelligent if I give breastmilk. It will increase bonding between mother and baby.” (A pregnant woman, had previous breastfeeding history, unemployed).
2. Women reported higher awareness of the benefits of breastfeeding.	“I heard that breastmilk should be given exclusively for six months. I will not add water or formula milk till my child is six months old as suggested. I think breastmilk alone has sufficient nutrients for my baby. I can also save money as I don’t need to spend on formula milk.” (A pregnant woman, had no previous breastfeeding history, unemployed).
3. Work as the main perceived barrier to practice EBF.	“Work is the main barrier. I sell fried rice and could take time off only 4–6 weeks after delivery. How could I breastfeed if there are a lot of customers.” (A pregnant woman, had previous breastfeeding history, street food seller). “I work in a private company and get 2 months’ maternity leave. It’s impossible for me to give breastmilk exclusively for six months.” (A pregnant woman, had previous breastfeeding history, employed).
4. Previous breastfeeding experience and concern for breastmilk supply play as influencing factors.	“I had difficulties in breastfeeding last time due to mastitis and very painful. If I suffer mastitis this time, I might add formula milk.” (A pregnant woman, had previous birth history employed). “Not sure whether I will have enough breastmilk because my breasts are small. This is my first pregnancy and hard for me to decide now. If I don’t have enough milk flow, formula milk will be an option.” (A pregnant woman, had no previous breastfeeding history, unemployed).
5. Health staff, family members and printed media were identified as the main sources of breastfeeding information.	“I receive breastfeeding information mostly from health staff, billboard, pamphlet and magazines. I love reading billboard especially if my favorite actress posts in it. My main information source is my sister who has experience in breastfeeding.” (A pregnant woman, had no previous birth history, employed).
6. Feedback on health staff as a source of information.	“I was always told to give breastmilk as it is good for baby by the doctors or the nurses when visiting antenatal clinic. I want to know more. I want to know… why we shouldn’t add water and what should I do, if baby cries or has hiccough or say, I don’t have enough milk. I saw others gave formula milk and seems their children are okay. Hospital staff are very busy and have limited time, I could not ask them (for more information).” (A pregnant woman, had previous breastfeeding history, unemployed).
7. Perceived self-efficacy to EBF.	“I think I could manage to breastfeed easily as I am not working and my husband supports me. My mother breastfed me when I was young and I am ready to feed breastmilk to my baby. I am confident.” (A pregnant woman, had no previous breastfeeding history, unemployed).

Regarding sources of knowledge, the majority of the respondents mostly described family members (mothers, mothers-in-law, sister), health staff and printed media (Table 3, theme 5). The internet as a source of knowledge was rarely mentioned. Though health staff were mentioned as a source of knowledge, women perceived that doctors and nurses from the hospital clinic were too busy to be asked questions (Table 3, theme 6). None of them claimed to have an issue concerning trustworthiness and reliability of the information they received. Evidently, if women said they had high confidence to practice EBF, they subsequently reported their intention to EBF despite challenges (Table 3, theme 7).

## Discussion

This study found that mothers’ intention to EBF was significantly associated with women’s working status, her knowledge level, household wealth index and obtaining knowledge from health staff or mobile internet. From the qualitative study, we found that returning to work was the main barrier to EBF; health staff and printed media were the most commonly reported reliable sources of information, and women with more knowledge about the benefits of breastfeeding had higher intentions to EBF than those with less knowledge.

This information is important because we have identified several key factors associated with women’s intention to EBF, which can help to target interventions to support EBF and the finding that women perceived work as a barrier to EBF is the important information to advocate the better support for breastfeeding in the workplace. Since the intention to breastfeed is a positive predictor for breastfeeding initiation and actual duration of breastfeeding [19–21], understanding these factors will help health policy makers or clinicians to develop better policies and programs to support EBF.

The main strength of the study was the use of a mixed method approach to understand the factors influencing pregnant women’s intention to EBF. This approach gave the researchers a greater depth of understanding about the factors associated with intentions to EBF, which would not have been captured by using a survey alone. For example, factors such as ‘unsure to practice EBF due to mastitis in previous breastfeeding’ and ‘printed media as a reliable source of knowledge’ were not found to be associated with women’s intention to EBF in the survey data analysis. Another important strength was our approach to minimize selection bias by randomly choosing pregnant women from hospital antenatal care waiting areas. Our selection of the largest public women’s hospital in Myanmar helped ensure we obtained a sample of women of various socio-economic backgrounds. To ensure a high quality quantitative data, we used digital data capture with a Commcare application [28] that reduced human errors with recording respondents’ responses and sped up data processing. By having a bilingual investigator (MPH), who translated the Burmese transcripts into English ensured appropriate translation that captured the detailed meaning of the participants’ words. This ensured that all the investigators could access the qualitative data and contribute to the qualitative analysis.

The main limitation was the cross-sectional design that prevented establishing causal relationship between the factors examined and women’s intention to EBF. Another possible limitation is that we failed to ask about ‘mobile internet as a source of knowledge’ in the survey although we identified this as an important information source for the women in the qualitative study. As the data was collected from only one hospital, we acknowledged that it could be a limitation for generalization of our study findings. The hospital selected for the recruitment, is the largest public hospital providing free quality delivery care service. It has a patient population with diverse ethnic and socio-economic backgrounds, with women presenting to the hospital from all over the country, including both rural and urban slum areas. Like our survey findings about work as the barrier to EBF, other studies have similarly reported that maternal work outside the home is a critical factor with a potentially strong influence on breastfeeding intention and duration [9, 12, 33]. Our qualitative study also revealed work as the major barrier to intentions to EBF and captured some additional information. For example, maternity leave in the private sector was found to be only 10–12 weeks and for a poor and self-employed woman; returning to work to earn a daily income might be more important than EBF, regardless of her awareness level. Other qualitative studies conducted in the United States [34] and Myanmar [35] have also highlighted that maternal work was the main barrier to practice longer EBF. Based on the study findings, we suggested to develop or enforce a more concrete social welfare policy in Myanmar, to help working mothers, through the provision of breastfeeding friendly facilities at both public and private sectors and enough maternity leave in the private sector. In Myanmar, maternity leave was legally increased from three to six months for government employees in 2014. As the policy does not apply to the private sector, more support from the private sector employers is needed. During interviews with two pregnant mothers from the private sector, short maternity leave was reported as a main challenge for EBF. ‘Providing a simple support as a breastfeeding room could contribute to the long-term productive capacity of your businesses’ is a message we think should be share with private sector employers. Several studies have reported that economic status is an influential factor for women’s intention to EBF and the duration of EBF. In our study, women from higher economic status had higher intention to EBF which is similar to other earlier studies [36, 37]. In contrast, some studies reported that women from low income families had higher breastfeeding duration, or income level did not have a significant role for women’s breastfeeding practice [38, 39].

To date, several studies reported that, in general, mothers with high breastfeeding knowledge and awareness levels have longer duration of EBF [9, 20, 21]. Similar to a study in Australia [12], our study found that breastfeeding knowledge level influenced women’s intention to EBF for six months. Our study revealed that particular sources of knowledge, such as health staff and mobile internet were found to be significantly associated with women’s intention to EBF. Un like a study in China, we did not find sources, such as printed media, families and friends were associated with intention to EBF [9]. In our study, health staff were found to be an important source of knowledge to support the women’s intention to EBF. A Cochrane meta-analysis from 29 countries suggested that support from health professionals increased the duration of breastfeeding up to the first 6 months postpartum [40]. It is worth noting that some women in our study claimed that health staff were too busy to be approached to get further breastfeeding information, although the women perceived the staff as a reliable source, similar to the interview findings from the Chinese study [9]. Our findings indicate that health care providers should provide enough breastfeeding related information to the pregnant mothers. Otherwise, as suggested by a literature review, if women have inadequate and inconsistent information, health staff support could have a negative impact on breastfeeding [41]. Health staff should spend more time to provide breastfeeding education and individual counselling during clinic visits but a mobile communication platform (mHealth) should be used as an additional channel of communications in promoting breastfeeding practice.

Little is known about the role of mobile internet in promoting breastfeeding in Myanmar. Myanmar had a low mobile phone ownership with only 2% mobile penetration rate in 2011 and earlier breastfeeding promotion studies in Myanmar did not focus on mobile phone related information. But mobile penetration has risen sharply since 2013, with 49% of adults in Myanmar owning a mobile phone in 2014 [42] and our study provides new and useful information for policy planners in Myanmar. Ethnicity and breastfeeding self-efficacy level variables were associated with women’s intention to EBF in the bivariate analysis (Table 1). Ethnicity was associated with breastfeeding duration in the United States study [36] and self-efficacy to breastfeed was associated with intention to breastfeed [11, 19, 43].

As the most commonly reasons given by respondents for their intentions to EBF were based on their perceptions of EBF could have positive effects on children’ physical health, breastfeeding educational context should be emphasized to the facts that maintaining EBF practice will have benefits for mothers’ health and children’s cognitive function. As our study examines intention to EBF before delivery, further research is needed to examine whether women with the intention to EBF for six months practice EBF after delivery.

## Conclusions

Intention to EBF amongst a sample of women in Yangon, Myanmar was influenced by occupation, household wealth, knowledge level and sources of knowledge from health staff or mobile internet. Breastfeeding promotion programs should target the poor, working women, and women with low breastfeeding knowledge, and health messages should be delivered via health staff and mobile internet. Facilities and support for breastfeeding at workplace should be improved.

## Abbreviations

EBF: Exclusive breastfeeding
WHO: World Health Organization

## References

[1] World Health Organization: Global Strategy for Infant and Young Child Feeding Report by the Secretariat. In*.* Geneva: World Health Organization; 2003.

[2] Duijts L, Jaddoe VW, Hofman A, Moll HA (2010). Prolonged and exclusive breastfeeding reduces the risk of infectious diseases in infancy. Pediatrics.

[3] Horta BL, Bahl R, Martines JC, Victora CG (2007). Evidence on the long-term effects of breastfeeding:systematic reviews and meta-analysis.

[4] Kramer MS, Kakuma R: The optimal duration of exclusive breastfeeding. In: Protecting Infants through Human Milk*.* edn.: Springer; 2004: 63–77.

[5] World Health Organization and The United Nations Children’s Fund: Ending Preventable Child Deaths from Pneumonia and Diarrhoea by 2025. The integrated global action plan for pneumonia and Diarrhoea (GAPPD). In*.* Geneva: World Health Organization; 2013.

[6] Brown CRL, Dodds L, Legge A, Bryanton J, Semenic S (2014). Factors influencing the reasons why mothers stop breastfeeding. Canadian Journal of Public Health.

[7] Chambers JA, Alder E, Hoddinott P, McInnes R (2007). A systematic review of measures assessing mothers' knowledge, attitudes, confidence and satisfaction towards breastfeeding. Breastfeeding Review.

[8] Binns CW, Scott JA (2002). Breastfeeding: reasons for starting, reasons for stopping and problems along the way. Breastfeeding Review.

[9] Jiang H, Li M, Yang D, Wen LM, Hunter C, He G, Qian X (2012). Awareness, intention, and needs regarding breastfeeding: findings from first-time mothers in shanghai China. Breastfeeding Medicine.

[10] Santo LCdE, de Oliveira LD, Giugliani ERJ: Factors associated with low incidence of exclusive breastfeeding for the first 6 months. Birth 2007, 34(3):212–219. 10.1111/j.1523-536X.2007.00173.x 17718871

[11] Dennis CL (1999). Theoretical underpinnings of breastfeeding confidence: a self-efficacy framework. J Hum Lact.

[12] Wen LM, Baur LA, Rissel C, Alperstein G, Simpson JM (2009). Intention to breastfeed and awareness of health recommendations: findings from first-time mothers in southwest Sydney China. International Breastfeeding Journal.

[13] Ministry of National Planning and Economic Development and Ministry of Health Myanmar: Myanmar Multiple Indicator Cluster Survey 2009–2010 Final Report. Nay Pyi Taw, Myanmar: Ministry of National Planning and Economic Development and Ministry of Health, Myanmar; 2011.

[14] United Nations Children's Fund: Infant and young child feeding database, 2015. http://data.Unicef.Org/nutrition/iycf.html. Accessed June 2016. In.

[15] World Health Organization: World Health statistics 2015. Part 1 health related millennium development goals. Geneva: World Health Organization; 2015.

[16] Black RE, Victora CG, Walker SP, Bhutta ZA, Christian P, de Onis M (2013). Maternal and child undernutrition and overweight in low-income and middle-income countries. Lancet.

[17] United Nations Children's Fund: Improving child nutrition: the achievable imperative for global progress. New York: United Nations Children's Fund; 2013.

[18] Forster DA, McLachlan HL, Lumley J (2006). Factors associated with breastfeeding at six months postpartum in a group of Australian women. Int Breastfeed J.

[19] Meedya S, Fahy K, Kable A (2010). Factors that positively influence breastfeeding duration to 6 months: a literature review. Women and Birth.

[20] Li R, Fein SB, Chen J, Grummer-Strawn LM: Why mothers stop breastfeeding: mothers' self-reported reasons for stopping during the first year. Pediatrics 2008, 122(Supplement):S69-S76. 10.1542/peds.2008-1315i 18829834

[21] Donath S, Amir LH (2003). Relationship between prenatal infant feeding intention and initiation and duration of breastfeeding: a cohort study. Acta Paediatr.

[22] Hmone MP, Li M, Alam A, Dibley MJ: Mobile phone short messages to improve breastfeeding feeding practices: a study protocol for a randomized controlled trial (M528) in Yangon, Myanmar. JMIR Research Protocols (in press). doi:10.2196/resprot7679. 10.2196/resprot.7679 PMC5508119 28659252

[23] Hmone MP, Dibley MJ, Li M, Alam A (2016). A formative study to inform mHealth based randomized controlled trial intervention to promote exclusive breastfeeding practices in Myanmar: incorporating qualitative study findings. BMC Medical Informatics and Decision Making.

[24] Hmone MP, Li M, Kingsley A, Alam A, Dibley MJ: Impact of SMS text messages on improving exclusive breastfeeding and reducing adverse infant feeding practices in Yangon, Myanmar: a randomized controlled trial. Submitted.

[25] World Health Organization: Indicators for assessing infant and young child feeding practices : conclusions of a consensus meeting held 6–8 November 2007 in Washington D.C., USA. In*.* Geneva: World Health Organization; 2007.

[26] Dennis CL (2003). The breastfeeding self-efficacy scale: psychometric assessment of the short form. Journal of Obstetric Gynecology & Neonatal Nursing.

[27] Filmer D, Pritchett LH (2001). Estimating wealth effects without expenditure data—or tears: an application to educational enrollments in states of india. Demography.

[28] Dimagi. In*.*; 2015. https://www.commcarehq.org/home/. Accessed September 2014.

[29] National Institute of Population Research and Training (NIPORT): Bangladesh Demographic and Health Survey 2011. Dhaka, Bangladesh and Calverton, Maryland, USA: Mitra and Associates, and ICF; 2013.

[30] World Health Organization: Indicators for assessing infant and young child feeding practices part 1: definitions. In*.* Geneva: World Health Organization; 2010.

[31] Rabe-Hesketh S, Skrondal A (2006). Multilevel modelling of complex survey data. Journal of the Royal Statistical Society: Series A (Statistics in Society).

[32] Braun V, Clarke V (2006). Using thematic analysis in psychology. Qual Res Psychol.

[33] Senarath U, Dibley MJ, Agho KE (2010). Factors associated with nonexclusive breastfeeding in 5 east and southeast Asian countries: a multilevel analysis. J Hum Lact.

[34] Holmes AV, Chin NP, Kaczorowski J, Howard CR (2009). A barrier to exclusive breastfeeding for WIC enrollees: limited use of exclusive breastfeeding food package for mothers. Breastfeed Med.

[35] Thet MM, Khaing EE, Diamond-Smith N, Sudhinaraset M, Oo S, Aung T (2016). Barriers to exclusive breastfeeding in the Ayeyarwaddy region in Myanmar: qualitative findings from mothers, grandmothers, and husbands. Appetite.

[36] Mitra AK, Khoury AJ, Hinton AW, Carothers C (2004). Predictors of breastfeeding intention among low-income women. Matern Child Health J.

[37] Persad MD, Mensinger JL (2008). Maternal breastfeeding attitudes: association with breastfeeding intent and socio-demographics among urban primiparas. J Community Health.

[38] Evans WD, Wallace JL, Snider J (2012). Pilot evaluation of the text4baby mobile health program. BMC Public Health.

[39] Turner C, Papinczak T (2000). An analysis of personal and social factors influencing initiation and duration of breastfeeding in a large Queensland maternity hospital. Breastfeeding Review.

[40] McFadden A, Gavine A, Renfrew MJ, Wade A, Buchanan P, Taylor JL, et al: Support for healthy breastfeeding mothers with healthy term babies. Cochrane Database Syst Rev 2017 (2). CD001141. 10.1002/14651858.CD001141.pub5 PMC6464485 28244064

[41] Dennis CL (2002). Breastfeeding initiation and duration: A 1990-2000 literature review. Journal of Obstetric, Gynecologic, & Neonatal Nursing.

[42] The World Bank: World Development Indicators. In*.*; 2016. http://databank.worldbank.org/data/reports.aspx?source=world-development-indicators. Accessed May 2016.

[43] Blyth R, Creedy DK, Dennis CL, Moyle W, Pratt J, De Vries SM (2002). Effect of maternal confidence on breastfeeding duration: an application of breastfeeding self-efficacy theory. Birth.

